# How Linear Tension Converts to Curvature: Geometric Control of Bone Tissue Growth

**DOI:** 10.1371/journal.pone.0036336

**Published:** 2012-05-11

**Authors:** Cécile M. Bidan, Krishna P. Kommareddy, Monika Rumpler, Philip Kollmannsberger, Yves J. M. Bréchet, Peter Fratzl, John W. C. Dunlop

**Affiliations:** 1 Department of Biomaterials, Max Planck Institute of Colloids and Interfaces, Potsdam, Germany; 2 Ludwig Boltzmann Institute of Osteology at the Hanusch Hospital of WGKK and AUVA Trauma Centre Meidling, 1st Medical Department, Hanusch Hospital, Vienna, Austria; 3 Materials and Processes Science and Engineering Laboratory (SIMaP), Grenoble, France; University of Notre Dame, United States of America

## Abstract

This study investigated how substrate geometry influences in-vitro tissue formation at length scales much larger than a single cell. Two-millimetre thick hydroxyapatite plates containing circular pores and semi-circular channels of 0.5 mm radius, mimicking osteons and hemi-osteons respectively, were incubated with MC3T3-E1 cells for 4 weeks. The amount and shape of the tissue formed in the pores, as measured using phase contrast microscopy, depended on the substrate geometry. It was further demonstrated, using a simple geometric model, that the observed curvature-controlled growth can be derived from the assembly of tensile elements on a curved substrate. These tensile elements are cells anchored on distant points of the curved surface, thus creating an actin “chord” by generating tension between the adhesion sites. Such a chord model was used to link the shape of the substrate to cell organisation and tissue patterning. In a pore with a circular cross-section, tissue growth increases the average curvature of the surface, whereas a semi-circular channel tends to be flattened out. Thereby, a single mechanism could describe new tissue growth in both cortical and trabecular bone after resorption due to remodelling. These similarities between in-vitro and in-vivo patterns suggest geometry as an important signal for bone remodelling.

## Introduction

Cells are not only sensitive to biochemical signals [1], but also to the mechanical properties [2] and the geometry [3] of their environment. They detect and respond to these physical characteristics at different length scales. On the sub-cellular level, cells sense and integrate mechanical information via their Focal Adhesions (FAs). These complexes of proteins link the extracellular environment to the cytoskeleton and enable cells to both apply and “feel” forces [4]. The internal cytoskeletal stress is constantly tuned by actin fibre remodelling and acto-myosin contractility [5], giving rise to a mechanical homeostasis in the cell [6]. This in turn enables the geometrical [7], [8] and physical [2] properties of the underlying extracellular matrix (ECM) or substrate to be sensed. The information is then transmitted to the nuclei [9] allowing cells to adapt proliferation [10], differentiation [11], apoptosis [12], spreading [13], migration [14], ECM production [15], and orientation during mitosis [16]. As cells are linked directly via cell/cell contacts or indirectly via the ECM, they can mechanically communicate with each other [17] and synchronise their individual decisions to act in a collective way giving rise to cell patterning [10], [18] and ECM organisation [19], [20] during morphogenesis for example [21].

At the tissue level, the influence and emergence of mechanical properties have been investigated in the context of cancer research [22], cardio-vascular disease [23] and tissue engineering [24]. While a lot of studies on porous scaffolds also revealed an effect of porosity and pore size on cell adhesion, proliferation and matrix deposition (see e.g. [25], [26]), relatively few focused on quantifying the role of scaffold architecture on tissue growth kinetics [27], [28]. In one study, Ripamonti and co-workers compared tissue growth in natural bone structures and artificial hydroxyapatite scaffolds implanted in vivo [29], and showed preferential tissue production in concave areas of the scaffolds, as also observed in vitro [30]. The kinetics of in vitro bone tissue growth was also measured in pores of controlled geometries in another study [27]. In their study, they showed that the thickness of tissue produced by osteoblasts depended on the local surface curvature. This led to the description of tissue development in terms of curvature-controlled tissue growth (CCTG), which gave good predictions of the tissue shape. Since this description is purely geometric, additional studies are required to elucidate the potential effects of mechanical and biological processes involved in the interfacial motion of tissue (e.g. cell proliferation and ECM production).

A classic example of the interaction between geometry and tissue growth can be found in the process of bone remodelling [31] which allows bone to renew and to adapt to slowly changing mechanical environments. During bone remodelling, three cell types are involved: osteocytes sense mechanical loads in existing bone [32], [33], [34]and forward the signal to osteoclasts which resorb old or damaged bone, and to osteoblasts which produce new collagenous tissue called osteoid. By definition, resorption and deposition are two processes that locally change the surface geometry of the bone tissue. In cortical bone remodelling, osteoclasts resorb bone, leaving cylindrical pores called osteons [35]. These are then refilled by osteoidal tissue with a central blood vessel, the Haversian canal [36]. During the remodelling of trabecular bone however, osteoclasts dig out small semi-circular channels or grooves called resorption pits or trails which can be seen as hemi-osteons, that are later refilled with osteoid by the osteoblasts [35]. Despite the continually changing local geometry, the mean curvature of the trabecular bone surface is tightly controlled [37]. Indeed, the signals responsible for such a precise spatial orchestration of the cells on the millimetre scale are still unclear. For instance, it is not clear why osteoblasts stop tissue production once a hemi-osteonal lacuna is filled. This provides a strong motivation to understand the influence of geometry on tissue deposition during bone remodelling – the goal of this paper.

Besides the quantitative description of tissue deposition on bone-like substrates, the present study proposes a new physical explanation of how the organisation of contractile cells leads to CCTG, as observed by Rumpler et al. [27]. Circular pores and semi-circular surfaces were designed in hydroxyapatite scaffolds to mimic osteons and osteoclastic resorption pits (hemi-osteons), respectively, and the scaffolds were incubated with MC3T3-E1 pre-osteoblast cells. In order to quantify geometry evolution on experimental images, a computational tool based on the approach of Frette et al. [38] was used to measure the curvature profiles and integrated into an algorithm for CCTG. In this paper, CCTG is shown to be equivalent to a simple geometrical construction representing the organisation of individual tensile elements and called the chord model. This new approach enables curvature-controlled tissue growth to be interpreted as the result of the superposition of linear elements such as stretched cells and collagen fibres. By comparing a simple geometrical model to experiments, this paper also highlights that the sum of mechanical and biological processes responsible for tissue growth responds to simple geometrical rules giving rise to the patterns observed in vitro. This suggests geometry as a key regulatory element for the tight control of tissue deposition during bone remodelling.

## Materials and Methods

### Production of the Hydroxyapatite (HA) Plates

**Figure 1:**
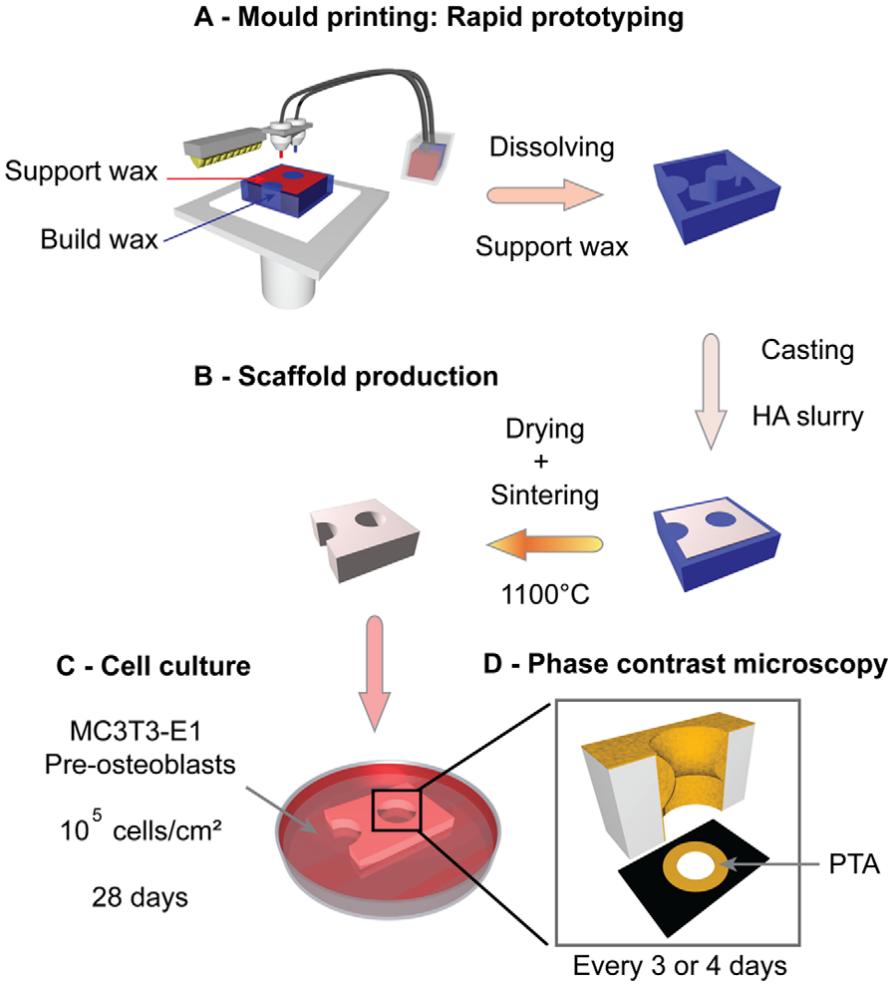
Experimental protocol. A - Moulds are produced by rapid prototyping. A build wax (blue) is used to print the mould in 3 D. A support wax (red) is added to reinforce the object while printing, and then removed by dissolution. B - Hydroxyapatite slurry is cast into the moulds, slowly dried and sintered. C - Pre-osteoblast cells are seeded (105 cells/cm^2^) on the scaffolds and cultured for 28 days. D - Tissue growth is quantified by phase contrast microscopy twice a week by measuring the projected tissue area (PTA) in each pore.

HA plates (2 mm thick) containing circular pores and semi-circular vertical channels (nominal diameter 1 mm) were made by slurry casting. The moulds were designed using the computer-aided design (CAD) software Alibre Design (Alibre Inc., Richardson, TX) and produced with a three-dimensional (3D) wax printer, Model Maker II (Solidscape Inc., Merrimack, NH) as described in [39] (Fig. 1A). The moulds were filled with a HA slurry made of 15 g of methacrylamide monomers (MAM), 5 g of N-N’-Methylenebisacrylamide (BMAM), 75 g of water and 12.5 g of Dextran for 300 g of HA powder, and crosslinked with ammonium persulfate and N,N,N’,N’-Tetramethylethylenediamine (TEMED). The structures were slowly air dried, pre-sintered and finally sintered as done in [40] (Fig. 1B).

### Cell Culture

Murine pre-osteoblastic cells MC3T3-E1 (provided by the Ludwig Boltzmann Institute of Osteology, Vienna, Austria) were seeded with a density of 10^5^ cells/cm^2^ on the surface of the HA scaffolds and cultured for 28 days in α-MEM (Sigma-Aldrich, St. Louis, MO) supplemented with 10% foetal calf serum (PAA laboratories, Linz, Austria), 0,1% ascorbic acid (Sigma-Aldrich, St. Louis, MO) and 0,1% gentamicin (Sigma-Aldrich, Steinheim, Germany) in a humidified atmosphere with 5% CO_2_ at 37°C (Fig. 1C).

### Imaging and Analysis

Each pore was imaged every 3 to 4 days using a phase contrast microscope (Nikon Eclipse TS100, Japan) equipped with a digital camera (Nikon Digital sight DS 2Mv) (Fig. 1D). All pictures were taken with a 4×objective, yielding the final image resolution 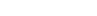.

The digital images were semi-automatically binarised using ImageJ (National Institutes of Health, Bethesda [41]). The contrast in the images enabled scaffold and tissue (black in the binarised images) to be distinguished from the medium (white).

### Measurement of the Tissue Production

Tissue production in the pores was quantified by determining the projected tissue area (PTA) formed in the pores (Fig. 1D). As this measurement is two-dimensional, it is only a proxy for quantifying the volume of growth into the depth of the pore. The free section of a pore, corresponding to the white regions in the binarised images, decreases with time. The PTA was then calculated by subtracting the binarised image at an initial time point from the image at the time of interest. As cells needed time to settle and start tissue deposition, the initial pore section was taken on the fourth day after seeding (D4).

The experiments presented here included 6 pores for each shape: circular pores (CIR) and semi-circular channels (SC). Two other sets of experiments repeated in the same conditions showed similar results (data not shown).

### Immunofluorescence Staining

Some scaffolds were washed with phosphate buffered saline (PBS), fixed with 4% paraformaldehyde and permeabilized with 0.1% Triton-X100 (Sigma-Aldrich, Steinheim, Germany). After 15 min blocking in 10% blocking reagent (Roche, Germany), the samples were incubated for 1 h in a 1∶200 solution of myosin IIb antibody (Cell Signaling Technology, Beverly, MA) and 1 h in a 1∶200 solution of anti-Rabbit IgG AF 488 (Cell Signaling Technology, Beverly, MA). Once washed in PBS, the tissue was stained for actin stress fibers by incubating with TRITC-Phalloidin 4×10^−8^ M (Sigma-Aldrich, Steinheim, Germany) for 40 min. After fixation, some of the samples were permeabilized as mentioned above and stained for nuclei with a 1∶300 solution of TO-PRO3 (Invitrogen, Oregon, USA) for 5 min. Images of stress fibres, myosin and nuclei were obtained using a confocal laser scanning microscope (Leica, Germany).

### Curvature Measurement

**Figure 2:**
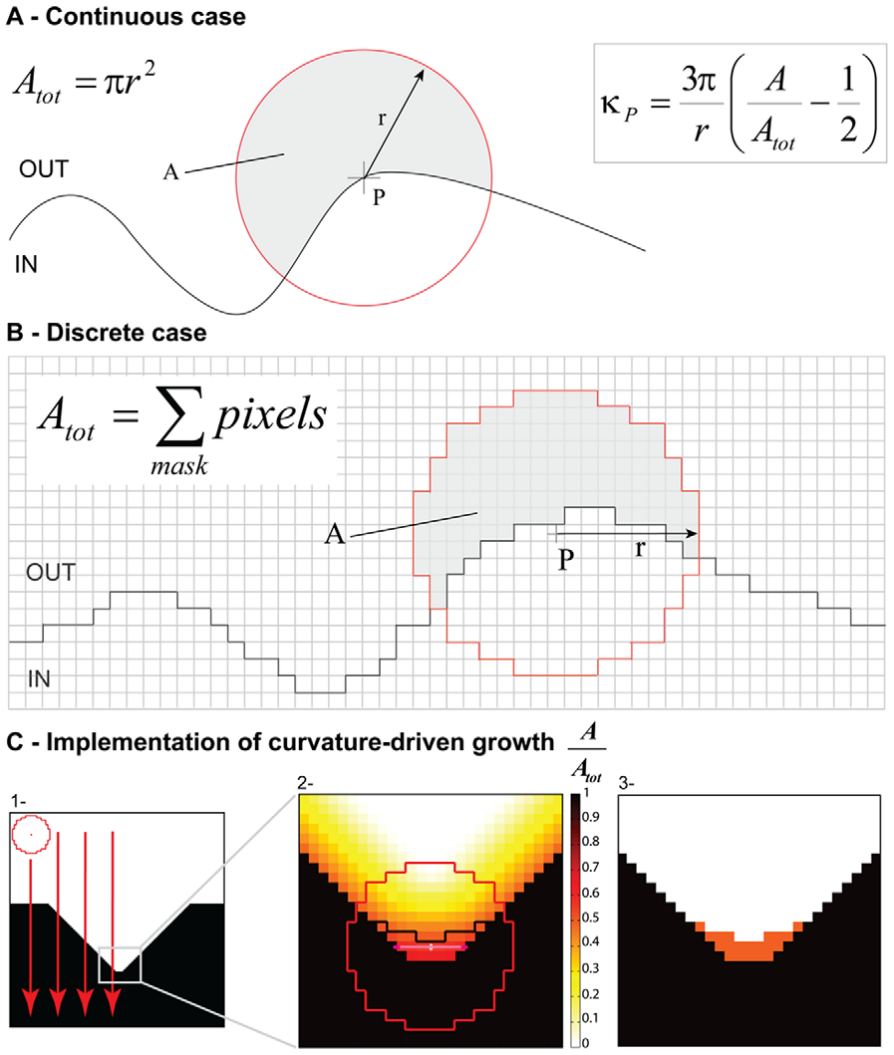
Computational methods. A-B - Principle of curvature measurement on continuous (A) and discrete (B) interfaces (adapted from [38]). The grey area represents the contributing area *A* in equation 1 where 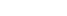 is the radius of the mask, 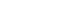 in A and 
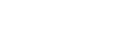
 in B. C - Implementation of CCTG. The whole image is scanned with the mask (1−) and the ratio 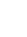 is attributed to each pixel (2−). A threshold of 0.5, corresponding to 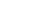 is applied: free pixels where 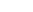 are filled with tissue (3−). The interface is then updated and the entire procedure is repeated.

**Figure 3:**
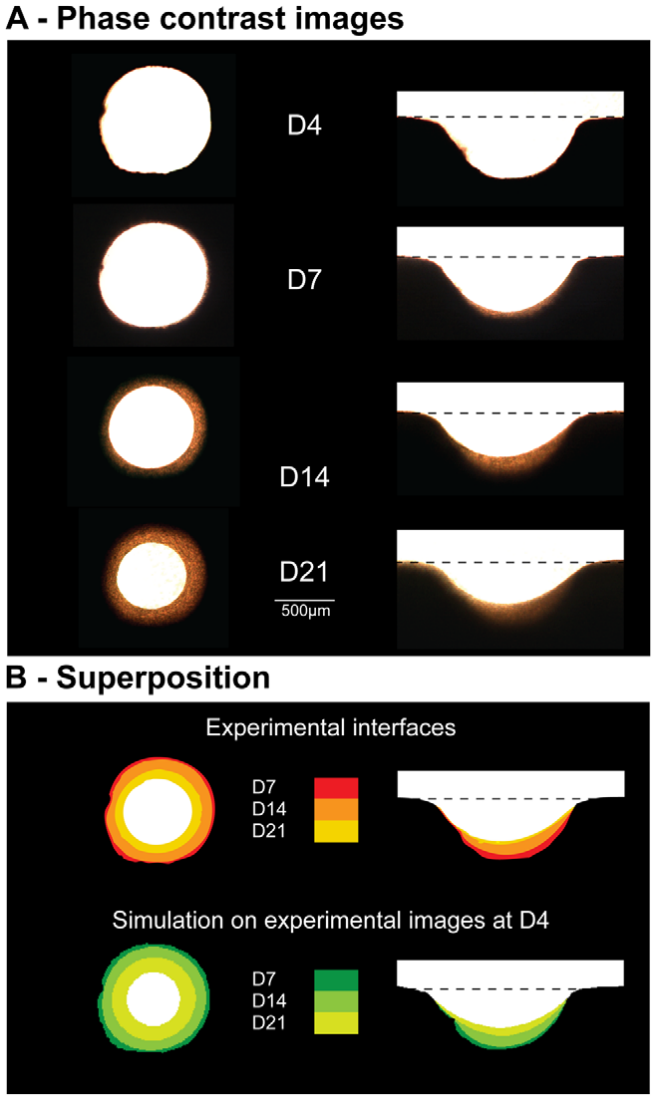
Qualitative results: evolution of the geometry. A - Evolution of the tissue interface in a circular pore and on a semi-circular surface. Images taken at different culture times (D4, D7, D14 and D21) during in vitro experiments show behaviours comparable to those observed in osteons and hemi-osteons during bone remodelling. B - The superposition of the interfaces obtained experimentally (top) compares to the one derived from CCTG applied to the actual geometry of the experimental pores at D4 (bottom). 7, 14 and 21 days of culture are simulated by 51; 170 and 289 steps for the circle and 34; 153 and 272 steps for the semi-circle respectively (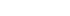 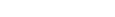).

The curvature profile of the interface between the tissue and the medium on each binarised image was calculated using Frette’s algorithm [38], [42] implemented in a custom made Matlab code (Matlab 7.8.0 R2009a, MathWorks, Natick, MA). This method has an advantage over other curvature measurements based on spline fitting [43], in that it can be applied directly to digital images coming from the phase contrast measurements. The algorithm first located the pixels on the tissue-medium interface in the binarised image. The local curvature 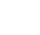 associated with an interface pixel was then estimated from the ratio of the number of black to white pixels lying within a given radius from the interface:
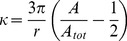
(Eq.1)where 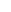 is the number of pixels in the mask and on the outer side of the interface, 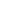 is the number of pixels in the mask and 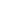 is the mask radius (Fig. 2A and B). The calculation was made for all pixels on the interface on each side of the border. The local curvature in one position of the interface was taken as the mean value of the curvatures measured on the outer pixel and the inner pixel. In the limit of a perfectly smooth interface and an infinitely small radius, this ratio corresponds to the local curvature. In this paper, a positive curvature is defined as a concave surface (Fig. 3). Average curvatures 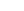 were determined along the perimeter of the pore for circles, and along a portion of the interface in semi-circles. The precision of the measurements are discussed in Text S1.

### Curvature-controlled Tissue Growth

An equivalent of the CCTG model presented by Rumpler et al. [27] was implemented by incorporating the curvature estimation of Frette et al. [38]. The technique to estimate interfacial curvature on a binary image was extended towards a description of growth by scanning the mask over the entire image, giving “effective curvature” values for all pixels (Fig. 2C). Assuming that growth occurs only in concave regions, each white pixel where the effective curvature is positive was changed to black, representing tissue deposition. The process was then iterated to describe CCTG. This method has the advantage that growth can be directly compared with the experimental pore geometries.

In the approximation 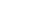 the local thickness of tissue produced in one step is proportional to the local curvature (for a proof see Text S1) and compares with the description of CCTG proposed in [27]:
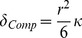
(Eq.2)


## Results

Tissue deposition was observed in each pore by phase contrast microscopy over a period of 28 days. Fig. 3A presents images taken at different times during the culture (D4, D7, D14 and D21) and is compared with the CCTG description in Fig. 3B. In circular pores, tissue deposition occured homogeneously along the interface, leading to a uniform concentric closing of the cylinder. On semi-circular channels, no tissue formed on the convex corners of the channel neither on the external flat surfaces. Growth is therefore pinned within the channel, resulting in different amounts of tissue as a function of position in the lacuna. In contrast to circular pores, the interface of semi-circular channels flattened with time.

**Figure 4:**
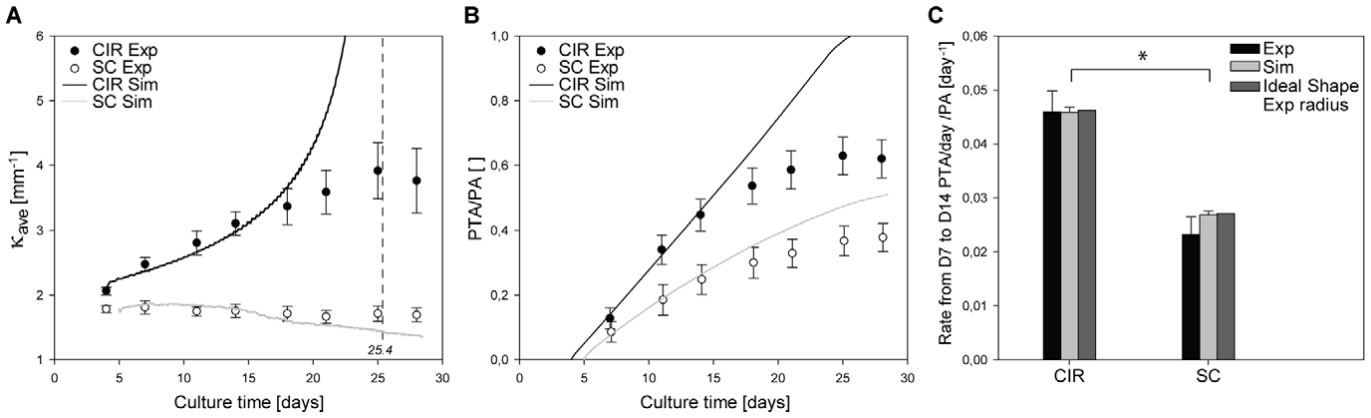
Quantitative results: curvature profile and growth rate. Quantitative analysis of tissue growth in circular pores (*CIR*) and on semi-circular channels (*SC*) of 1 mm diameter. A - The average curvature along the perimeter of the circular pore and on a given portion of the semi-circular surfaces is measured on experimental images at different culture times. As 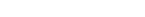 theoretically circular pores should be filled in about 432 steps or 25.4 days. B - The projected tissue area (PTA) is normalised by the area of the pore (PA) at D4 (reference) and reported as a function of culture time. In A and B, the full lines correspond to the prediction given by CCTG (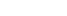; 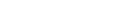). A lag time is used to overlap simulated and experimental data (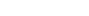 and 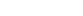). C - Growth rates are calculated between D7 and D14 with the experimental and the simulated data as well as data simulated on ideal geometries with a radius derived from the experimental images. ANOVA analysis shows no significant differences between the methods used but a statistical difference in the tissue growth rates achieved in CIR and SC (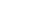). Dots and error bars represent mean values and standard errors, respectively (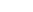).

The evolution of tissue shape reveals the determining role of the boundary conditions in the interfacial motion between 4 and 28 days. The average curvature measured on the experimental images increased with ongoing tissue growth in circular pores, whereas curvature slowly decreased on a semi-circular channel (Fig. 3A and 4A).

**Figure 5:**
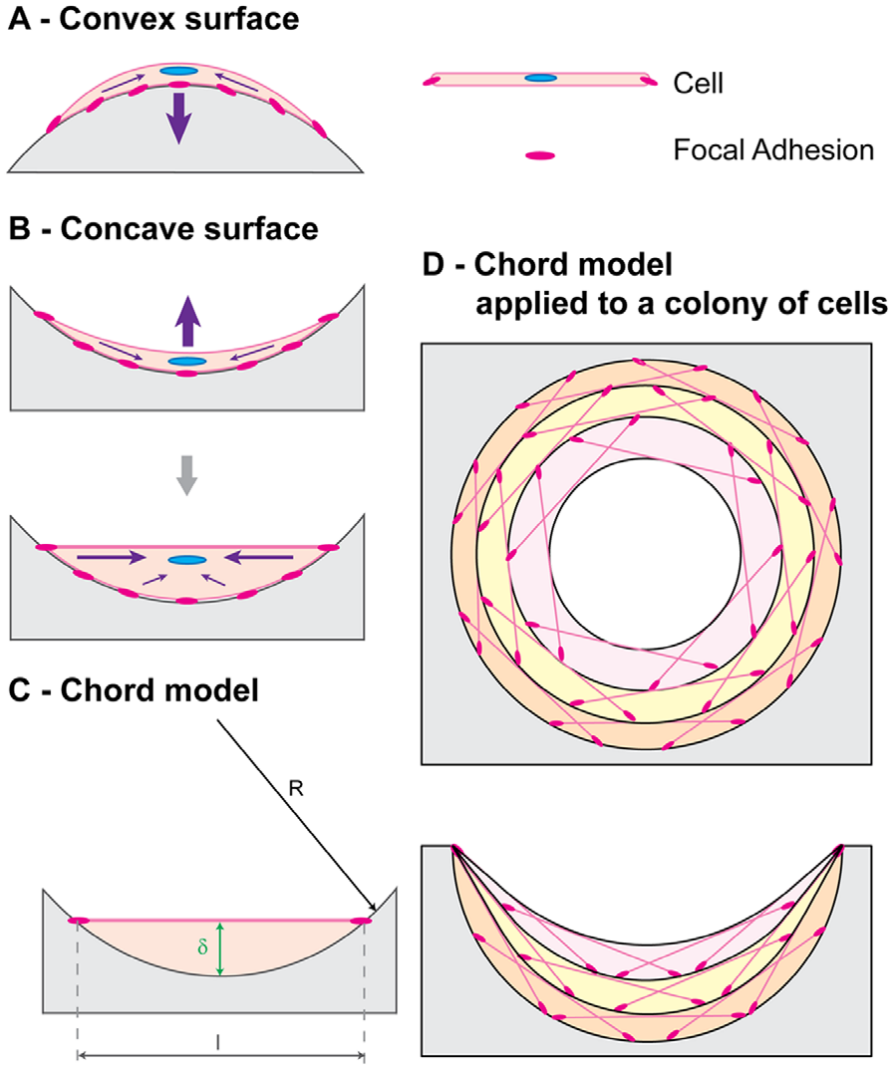
A chord model to describe tissue growth. After adhering on a substrate (pink dots), a cell contracts its cytoskeleton (purple arrows) to reach a stable tensile state. A - On a convex surface, the cell remains bent and exerts pressure on the substrate. B - On a concave surface, cell contraction stretches the membrane and results in a local flattening of the surface. C - A chord representing a static stretched cell defines an element of tissue, which thickness 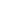 is proportional to the local curvature of the surface. D - A collection of stretched cells sitting on a concave surface can be seen as an assembly of segments. Each cell locally generates a zero curvature and defines an element contributing to the local thickness of tissue produced. With this new interface being defined, another collection of cells can settle and contribute to tissue growth. The interfacial motion derived from this simple geometrical interpretation compares with the experimental observations (Fig. 3).

On the growth curves in Fig. 4B, the PTA was normalised by the area of the pore (PA) measured on the fourth day of culture. In semi-circular channels, PA was taken to be the free area under the scaffold surface (dashed line in Fig. 5). A direct comparison of the fraction of available space filled with tissue was then possible. The experimental data displayed a linear increase of the amount of tissue produced in circular pores up to day 14 in agreement with previous results [27]. Afterwards, tissue amplification slowed down. Comparison of the early growth behaviour in circular and semi-circular channels, calculated between day 4 and day 14, revealed that tissue growth was significantly higher in the circles compared to semi-circular surfaces (Fig. 4C).

Although the local curvature was supposed to be the same on each point of the surface, the initial growth rates (between D4 and D14) in a circular pore and on a semi-circular surface are different (Fig. 4C). A two-way analysis of variance ANOVA showed a statistical difference between the shapes (CIR vs SC) and no significant influence of the methods used (Experiment vs Simulation on experimental shapes vs Simulation on ideal shape). All pair-wise multiple comparisons were done following the Holm-Sidak method and 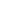 values of less than 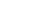 were considered significant. The geometry of the substrate influenced the speed of tissue production by the cells. The CCTG description correctly predicted that the average curvature diverges as the circular pore filled whereas it should converge toward zero (flat surface) in semi-circular channels (Fig. 4A).

In order to compare predictions and in vitro results, a time scale parameter 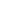 in 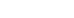 was derived from the ratio between simulated and experimental growth rates measured in circular pores in 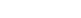 and 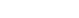 respectively. The tissue growth rate was derived from the simulations performed on experimental images with 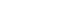, 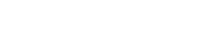 and experimentally measured on the early stage (D7 to D14) and is considered constant: 
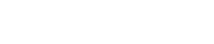
 The time scale used in the following is thus 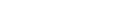


Quantitative results predicted by application of CCTG on experimental images at D4 are reported in Fig. 4B. The simulated projected tissue areas (PTA) were normalised by the area of the respective pores at D4 (PA) and averaged 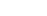 An additional lag time was used to overlap simulated and experimental data: 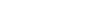 and 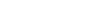 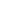 represents the time cells need to spread and colonize the scaffold before starting growth and is known to depend on the geometry of the pore [44]. Once the single free parameter of the calculations is fitted on the experimental growth in circular pores 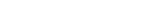 CCTG also correctly described the initial growth behaviour on semi-circular channels (Fig. 4A, B and C). However, it did not explain the notable slowdown observed experimentally in both geometries from D14.

### A “Chord Model” to Explain Curvature-controlled Tissue Growth

Although CCTG can predict both the geometry and the linear kinetics of tissue formation, it contains no intrinsic time scale and provides no mechanistic explanation of the curvature sensing of cells and tissue.

In the following, tissue is considered as a collection of stretched cells and fibrous ECM, and growth is described as occurring via the assembly of such tensile elements (chords) on a surface. In this section, it is demonstrated that CCTG is a direct consequence of this simple geometric construction. Besides giving a mechanistic interpretation of the CCTG on the cellular level, the chord model also motivates the interaction range (mask size) chosen for measuring curvature and thereby justifies the time scale of the computational implementation.

As cells are the tissue manufacturers, a geometrical description of single cells settled on a surface (Fig. 5) provides hints to the local dependence of tissue organisation on the geometry. Once attached to the substrate, cells contract their cytoskeleton thus defining a new interface between the FAs [45]. If the surface is flat or convex, cytoskeletal contraction results in a downward motion of the cell towards the substrate (Fig. 5A). However, if the surface is concave, the contracting cell is stretched between the FAs and locally forms a flat interface (Fig. 5B).

The chord model presented here consists of a tensile element of length 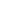 that connects two points on a surface (Fig. 5C) and locally defines a new interface. The effect induced in the perpendicular direction can be described using the largest distance 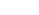 between the chord and the substrate. Simple geometrical relations (detailed in Text S1 and Figures S1 and S2) demonstrate that the local interfacial motion induced by the deposition of a single chord 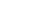 is proportional to the local curvature 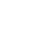 with the hypothesis 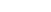:
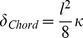
(Eq.3)


Combining this effect for all possible positions of the chord on the substrate predicts the location of the interface once a collection of tensile elements has been laid down (Fig. 5D). Additional layers can then settle iteratively on the surface.

Equations 2 and 3 demonstrate that the superposition of tensile elements on a curved surface generates an interfacial motion equivalent to the CCTG evolution presented earlier (Fig. 2 and [27]). Using 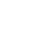 as the radius of the mask in the computational method leads to full quantitative consistency between the chord model and the CCTG description: 
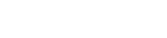
 Mathematical details are provided in the Text S1. Moreover, Fig. 5D reveals that geometries derived from the chord model also compared well with the experimental observations (Fig. 3).

**Figure 6:**
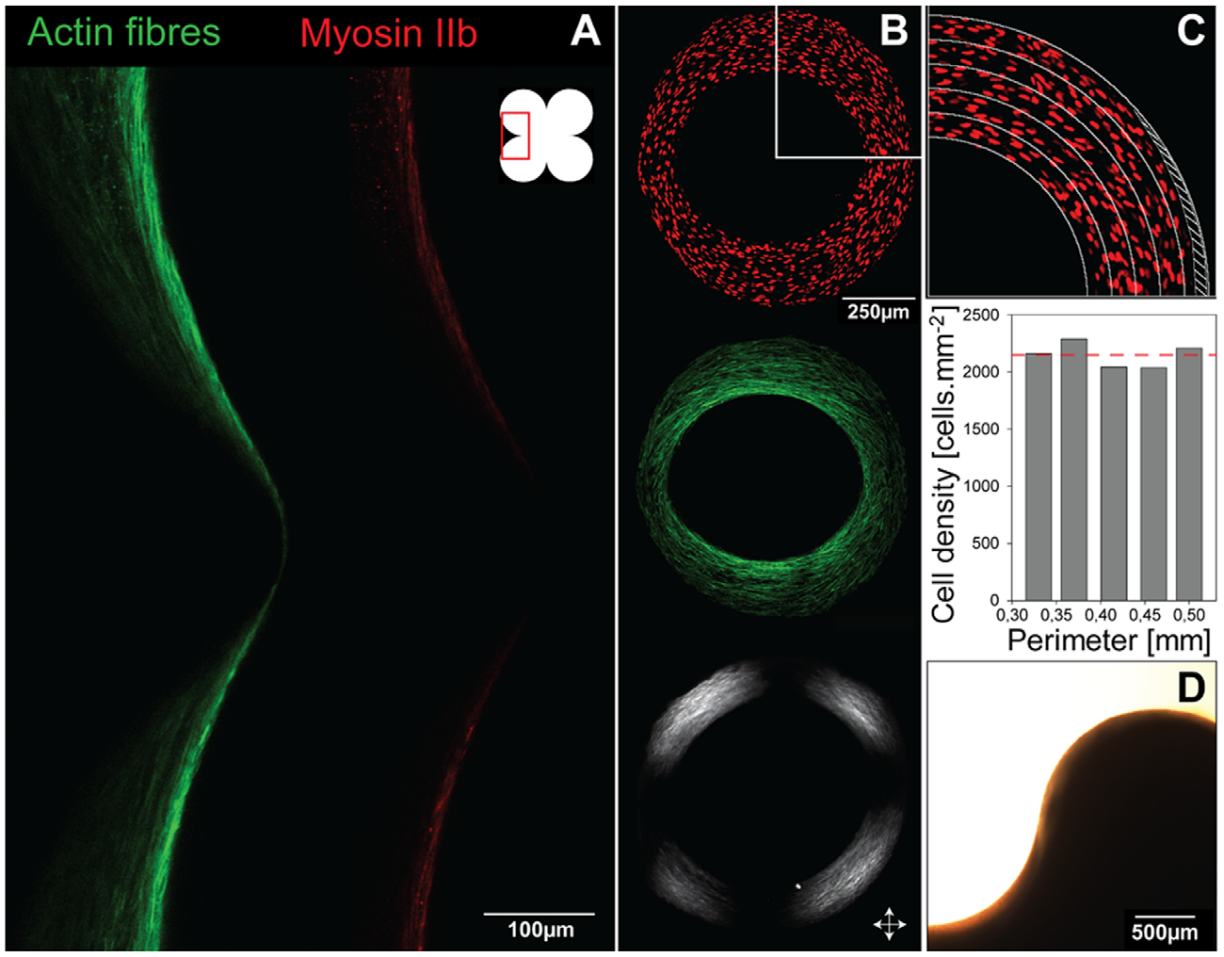
Tissue organisation. A - Tissue produced in a pore made of 4 adjacent circles and stained for actin stress fibres and myosin IIb. Actin fibres colocalised with myosin IIb are present on the whole surface but their higher density on concave interfaces suggests a local higher stress state of the cells. B - Tissue is made of cells and collagen. Nuclei (*red*), actin stress fibres (*green*) and collagen fibres (*visualized by polarized microscopy*) are oriented parallel to the interface. The white arrows show polarisation direction. C - The homogeneous distribution of nuclei shows that cell density is independent of geometry and suggests a local dependence of cell proliferation on the local curvature. D - An example of a convex HA surface (D35) on which only a mono-layer of tissue was formed.

Considering cells as tensile elements, the chord model can describe tissue growth and its curvature-driven behaviour. Additional support comes from the observation of actin fibres that indicate stresses produced by interacting cells. These stress fibres formed rings inside circular channels (Fig. 6B), as previously observed [27], which are reminiscent of contractile actin –myosin rings found in wound healing for other cell types [46], [47], [48]. Actin fibres co-localizing with myosin are shown in concave regions on Fig. 6A. These fibres have an arrangement very similar to the chords in Fig. 5B, supporting the idea that cells in the tissue collectively exert tensile stress as they adhere to matrix and substrate. On convex surfaces however, Fig. 6A clearly shows a much lower density of contractile chords, also in agreement with the model in Fig. 5. A convex surface (Fig. 6D) was also tested and interestingly only a mono-layer of tissue was observed even up to late growth stages.

Staining tissue for cell nuclei reveals a homogeneous cell density all over the projected tissue area (Fig. 6C). This showed that cell density is independent of curvature. Note that the global geometry of the new interface was independent of the number of chords, i.e. cell density, in one layer. Moreover, the geometry of a substrate is known to influence cell proliferation by determining the stress distribution in the contractile cell layer [10]. Although no proliferation study was performed here, the constant cell density suggests that cell proliferation adapts as the curvature increases and the adhesion surface decreases during tissue growth, leading to the overall constant cell density.

In the computational description presented earlier, the radius of the mask defined the interaction range around a given point and influences the precision of curvature estimation. The equivalence between the CCTG and the chord model together with the cellular approach proposed above motivated us to scale this range to the approximate length of a cell: 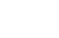 For an elongated osteoblast, 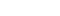 but with 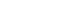 and 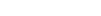 the actual cell length considered here is 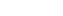 Thereby, the effective curvature values derived in the computational implementation of CCTG represent what cells “feel” from the geometrical features of the surface.

In terms of PTA, the simulated growth rate in 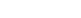 in an ideal circular pore is constant:
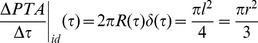
(Eq.4)


This constant rate derived in closed convex shapes (circles) confirms the equivalence between the chord model and the CCTG description proposed by Rumpler et al. [27], see also Fig S3.

According to equation 4, the simulated growth rate and thus the time scale of the model depend on the interaction range chosen. The simplest approach to determine this time scale is to assume that the experimental growth rate is also proportional to curvature. This requires the definition of one parameter 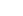 that fits the time 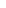 of simulated growth in steps to the time 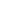 of experimental tissue growth in days, which leads to:
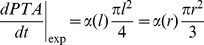
(Eq.5)


Equations 4 and 5 show that 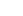 scales with the inverse of the square of the interaction range chosen in the model and can always be derived by comparing simulated and measured tissue growth rates in the experimental pores. The interaction range being fixed to 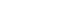, 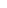 is a constant accounting for the kinetics of all the biological phenomena contributing to tissue deposition (cell migration, proliferation, ECM synthesis, etc.).


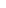
 and 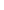 being proportional, the interfacial motion derived from the model can be described as a continuous function of time 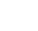 To quantify kinetics, the evolution of the distance between tissue interface and the substrate (in 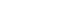) was derived in an ideal pore by integrating equation 3:
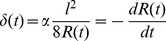
(Eq.6)


This gives a solution in terms of curvature:
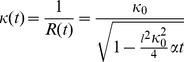
(Eq.7)


The time needed for an ideal circle to be filled was determined for a radius equal to zero and an infinite curvature:
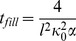
(Eq.8)


## Discussion

The amount and the shape of the tissue produced by MC3T3-E1 cells cultured in pores of controlled geometries were quantified in terms of PTA and curvature on phase contrast images taken over a period of 28 days. The chord model not only agrees with the computational implementation of CCTG as described in Fig. 2, but it also provides a relevant interpretation on the cellular scale of the equivalent behaviour observed during tissue growth (Fig. 3). Moreover, the works of Théry et al. [13] about the shape and the stress state of a cell after spreading and contraction, support the approach sketched on Fig. 5.

As described earlier, one step of the computational implementation of CCTG represents roughly the contribution of one layer of cells to the tissue thickness. In agreement with the hypothesis of CCTG, the simulation predicts a constant tissue growth rate (in 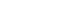) in an ideal circle [27]. This rate only depends on the cell size, arbitrarily set to 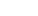 (Eq.7). Measuring the initial experimental rate (in 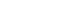) in circular pores enabled us to fit the model of growth with a unique parameter that introduces a linear time scale by giving the number of steps needed to represent one day of experiment: 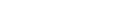. Although this parameter should theoretically represent the number of cell layers deposited in one day of culture, the high value suggests that some assumptions are too simple. For example, stretched osteoblasts in culture are probably not homogeneous in size 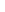 and are likely to be larger than 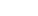. Moreover, a chord model only based on cells implies that the contribution of the ECM is neglected although Fig. 6B reveals the presence of collagen fibres aligned with the interface, just as actin fibres in stretched cells. Considering larger cells and/or adding collagen fibres in the definition of the tensile elements would increase the simulated growth rate and decrease 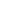 toward more realistic values. Importantly, 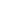 does not interfere with the geometrical behaviour of the interface but just rescales the evolution in time.

The circular pores and semi-circular surfaces produced in the experiments were chosen with the same radius, i.e. the same local curvature, along the interface. Although CCTG supposes a local growth rate proportional to the local curvature, Fig. 4C shows a significant difference in the normalised global growth rates (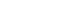) on circular pores and semi-circular surfaces. The qualitative results (Fig. 3) as well as the evolution of the average curvature (Fig. 4A) suggest the importance of the boundary conditions for the pattern of tissue deposition. As no growth occurs on the convexities (Fig. 6D), the tissue laid down within concavities flattens the surface in semi-circular channels, i.e. decreases the average curvature. In circular pores however, the concentric growth increases curvature.

**Figure 7:**
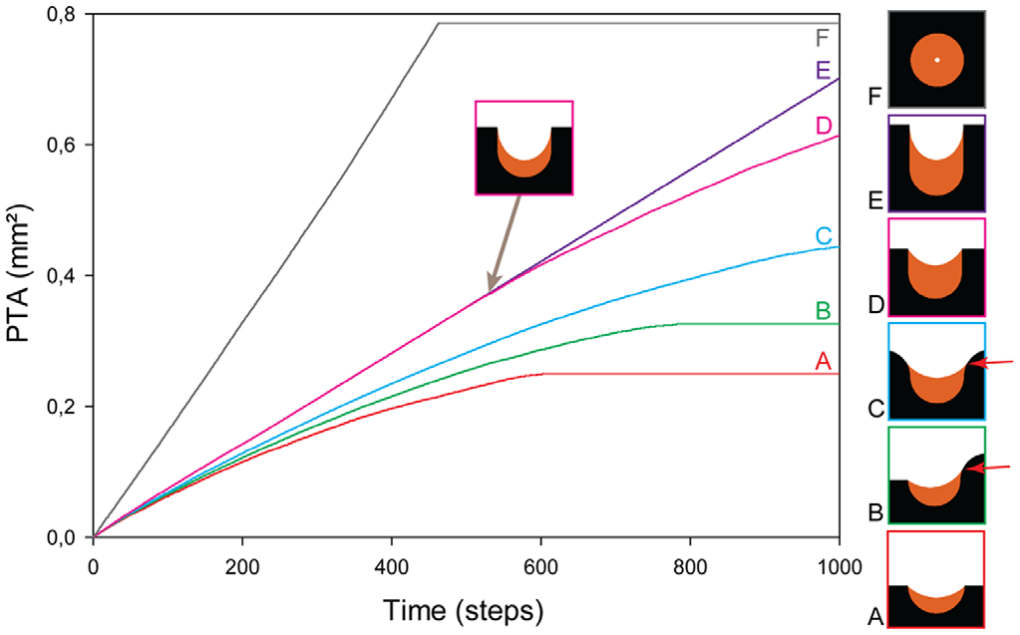
Importance of boundary conditions. Tissue growth (orange) is simulated on different artificial images using the CCTG description (A to F). The predicted PTA is reported as a function of iteration steps. Each initial interface (black) contains a semi-circle with a radius of 0.5 mm. The different boundary conditions show the influence expected on tissue growth rate and organisation. On A, B and C, the model predicts that the sharper the convex corners, the slower the growth. Tissue is eventually deposited on convex surfaces after the surroundings have been filled and the interface has locally become concave (red arrows). Comparing A, D and E reveals that shifting the convex corners upward prolongs the duration of a constant growth rate which is half of the one obtained in a full circle (F). Tissue deposition can expand on the walls until it reaches the convex corners. From this time point (inset), the surface joining the pinning points is minimised, which decreases the curvature and slows the growth.

To underline the determining role of the convex corners as seen in Fig. 6, CCTG was simulated on artificial images (Fig. 7A–F). All geometries were based on a semi-circle (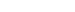) that is differently linked to the surrounding flat surfaces. Fig.7 shows that although the local curvature is the same on a given portion, tissue deposition (in time and space) depends strongly on the geometry of the surroundings. As the tissue grows, changes in the curvature profile of the interface affect both local and global growth rates. Although the phenomenon is slightly exaggerated due to the discrete character of the computational method (Fig. 7A and B), it is interesting to note the slowdown of growth when tissue reaches the convex corners. This suggests geometry as a potential signal for osteoblasts to decrease and eventually stop tissue production when a hemi-osteonal lacuna is filled.

Fig. 4B reveals a slowdown of the experimental tissue growth after 18 days of cell culture that is not predicted by the chord model fitted with a linear time scale. However, more complex scaling laws could be used to depict the non-linearities induced as cells slow down proliferation and ECM synthesis when they differentiate and mature [49]. For example, pre-osteoblasts differentiate towards osteoblasts during culture and begin to synthesise alkaline phosphatase (ALP). As the plateau often appears after 14 days of culture or later, which approximately corresponds to the beginning of ALP synthesis by such cells [50], the influence of the differentiation could be a possible explanation for the decrease in tissue production. In parallel, the ECM synthesized by the osteoblasts also undergoes maturation, whereby cross-link formation in the collagen matrix increases with culture time [51], [52]. This could implement a denser packing of the tissue and explain the plateau in PTA. As the CCTG description is intrinsically a geometrical description, adapting the time scale would be a simple way to take the effects of cell and matrix maturation into account. For a given interaction range, the number of steps representing one day of culture 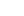 would then decrease with time, and the scaling law would require an additional time characteristic representing the slowdown of cell activity with ageing.

Alternatively, the plateau in tissue production observed after two weeks of culture may have a geometrical origin. In the experiments, tissue is grown in 2 mm thick scaffolds with straight sided pores, and only the projected tissue area is measured on phase contrast images. Using PTA as a proxy to quantify the amount of tissue produced in the pore implies that the local tissue thickness is homogeneous along the third axis, which is unlikely. Indeed, cells need time to migrate and therefore can not build the same thickness of tissue simultaneously throughout the depth of the channel. Moreover, the extremities of the pore present convex corners in 3 D and such boundary conditions are expected to affect the growth pattern along the z-axis. As a consequence, pores are unlikely to remain straight during growth, and a second principal curvature (different from zero) should then complete the geometrical characterisation of the interface in 3 D. Although this second principal curvature is expected to play a role, the approaches proposed in this study assume that only one principal curvature (in the image plane) changes during growth whereas the other remains constant and zero (straight sided pores). The slowdown of tissue growth observed in terms of PTA could then be explained by the emergence of a convexity (negative second principal curvature) in the z-direction that is not taken into account in the previous calculations. Extending the CCTG description to 3 D would be of great interest to understand which combination of the two principal curvatures is relevant for tissue growth: mean curvature, Gaussian curvature, maximal curvature, etc. As such models predict interface evolution toward surfaces of minimal energy, this 3 D mean curvature would then decrease and tend towards zero, much akin to what is observed in trabecular bone [37].

Interestingly, curvature-controlled growth is well known in physics and material sciences and has been used to describe electrochemical coating [53]–[54], solidification [55], and grain growth [56], for example. Such processes come about in systems with high surface tensions, in which surface energy is linked to curvature through the Laplace equation, as commonly seen in wetting problems [57]. Surface tension has also been shown to be a determining factor in biology, mainly in the context of the Differential Adhesion Hypothesis [58]. This interfacial characteristic is not only responsible for self sorting on the cell level during gastrulation [59] and tumour invasion [60] but also for tissue organisation [28].

While it was known that tissue-producing cells respond to geometry [29] following a principle of CCTG on a millimetre scale [27], the present study shows that the patterns of growth obtained in circular pores and on semi-circular channels are analytically equivalent to those derived from a simple construction based on tensile elements representing stretched cells. No direct geometry sensing is necessary to explain the resulting curvature-controlled growth. The shape of the surface affects the spatial distribution of FAs and thereby the shape of the contractile cells [13] as well as the forces they sense [8] and produce [10]. Adding a time scale enables the model to predict the kinetics of tissue deposition: faster growth occurs in circular pores compared to semi-circular surfaces.

The chord model was able to explain the shape-dependence of growth solely in terms of tension and curvature, without any biological mechanisms such as stress-dependent proliferation or migration. Although such mechanisms are involved on the cellular level and need to be taken into account in a physiological context, our results show that the interplay of contractility and geometry alone can coordinate growth in scaffolds. This reveals a generic physical control mechanism for biological growth processes in bone, independent of specific functional aspects and signalling pathways, that may also be relevant to other tissue types.

The interfacial motion predicted by the model and supported by the experiments is similar to the one occurring in osteons and osteoclastic resorption lacunae during bone remodelling: while circular pores are filled in a concentric way, semi-circular channels are filled layer by layer until the interface becomes flat i.e. the curvature of the surface becomes zero. This implies that osteoblasts do not need a specific signal to stop matrix production when the resorption pit is filled, but the gradual flattening of the bone surface during the filling process are sufficient as a cue. Interestingly the observation that semi-circular pores fill at a slower rate than circular ones is also observed in trabecular and cortical bone, with the filling of hemi-osteons being slower than for osteons (see e.g. [61], [62], [63]). These results strongly suggest that surface geometry is an important signal for controlling bone remodelling. In this respect the model may also have implications for tissue engineering and of course may be interesting to use it in the design of scaffold materials for implants [64], [65], [66]. One major difficulty in testing the model in-vivo is the limited amount of kinetics data available in which local growth rates within a scaffold have been measured. It is possible that recent developments in *in-vivo* CT may provide suitable data that enables a comparison with the model [67].

The chord model presented in this paper makes the link between the macroscopic curvature-controlled tissue growth observed in vitro and in vivo, and the assembly of stretched cells and other fibrous elements making up the tissue.

## Supporting Information

Figure S1 **Descriptive scheme of the local geometrical configuration in a continuous case.**
(TIF)

Figure S2 **A simple geometrical construction can explain the evolution of the interface observed in in-vitro experiments.**
(TIF)

Figure S3
**The equivalence of the CCTG description to the chord model if the radius of the mask is** 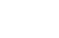 The interfaces derived from both models after 100, 200 and 300 iterations are shown as coloured lines for CCTG and grey lines delimiting coloured regions for the chord model (the radius 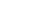 being calculated analytically with 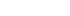 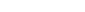).(TIF)

Text S1 **A discussion of the precision of curvature measurements on digital images and a demonstration of the equivalence between the chord model and curvature driven growth.**
(DOC)
