# Supplementary material for: How Linear Tension Converts to Curvature: Geometric Control of Bone Tissue Growth
Source: PLoS One. 2012 May 11;7(5):e36336. doi: 10.1371/journal.pone.0036336 (PMC3350529; doi:10.1371/journal.pone.0036336)
Supplement: Text S1 — A discussion of the precision of curvature measurements on digital images and a demonstration of the equivalence between the chord model and curvature driven growth. (DOC) [file pone.0036336.s004.doc]

**Precision of curvature measurements on digital images**

Curvature is defined in a given point by the second derivative of the parameterised curve. The method used in this study and applied to pixellated images can thus only provide an estimation of the local curvatures. The precision of the measurement depends on both the resolution of the image ($1mm=205pxl$) and the size of the circular mask. As $2r$ needs to be odd, the radius is taken to be $r=8.5pxl$, which involves an error of 0.9% on the area of a digital mask with respect to an ideal one. To estimate the precision of the curvature measurements, tests are performed on circles with different radii at different resolutions (data not shown). For a given scale, the average curvature measured on each circle is proportional to the inverse of the radius in pixels (analytical curvature). If $1mm=205pxl$, the error on the average curvature stays under 2%.

**The computational implementation of curvature-controlled tissue growth
and the chord model are equivalent**

# The computational implementation of curvature-controlled tissue growth (CCTG) based on the method of Frette et al.

The effective curvature in centre of the scanning mask (radius $r$) is given by: $\kappa=\frac{3\pi}{r}\left(\frac{A}{A_{tot}}-\frac{1}{2}\right)$ (Eq.1)

The position of the interface where the effective curvature is zero is then given by $\delta\left(r,R\right)$ when $\frac{A}{A_{tot}}=\frac{1}{2}$.


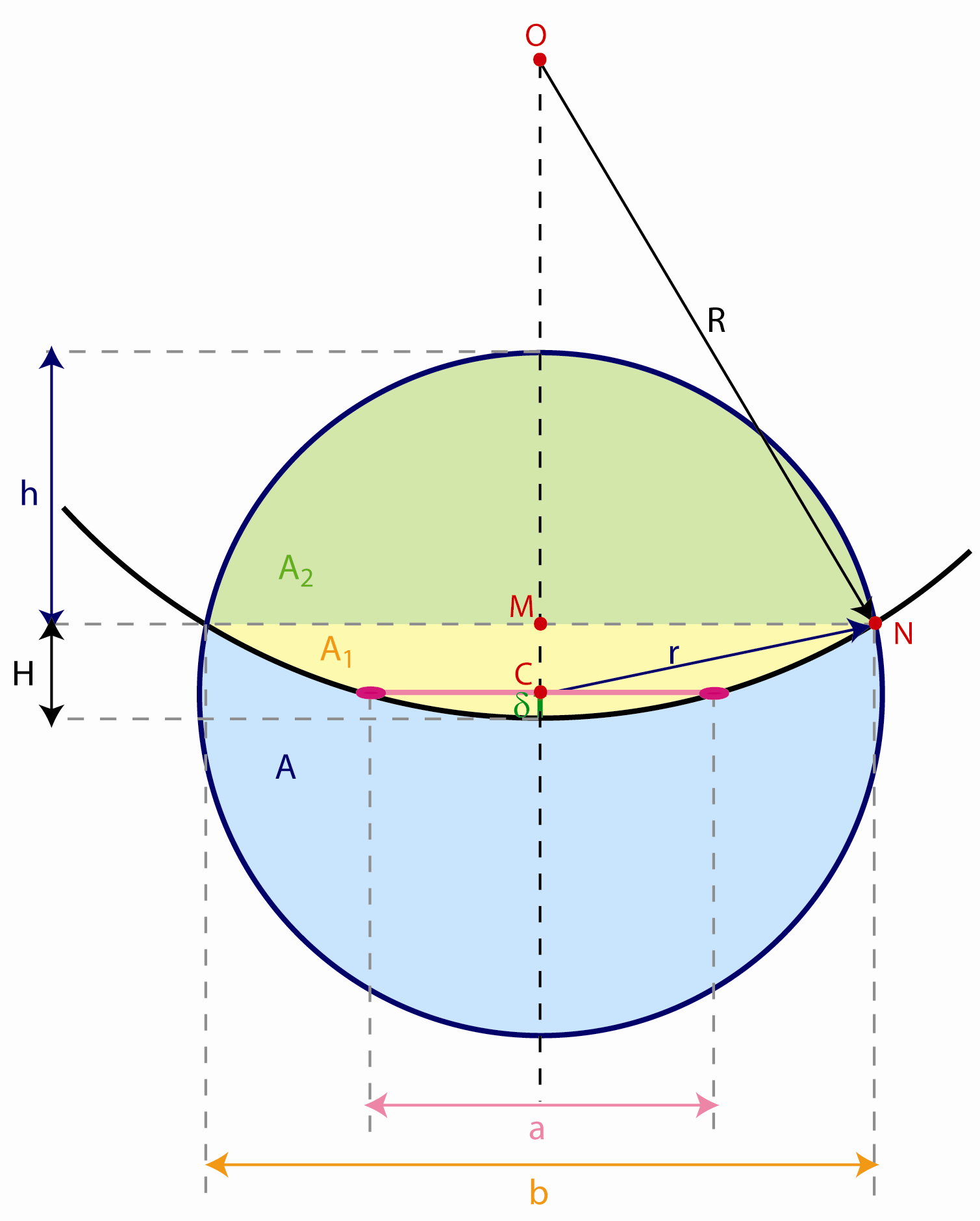


FIGURE S1.

Descriptive scheme of the local geometrical configuration in a continuous case.

The large circle portion represents the local interface ($R$,$H$) and the small circle represents the mask ($r$,$h$).

$A=\pi r^{2}-\left(A_{1}+A_{2}\right)$ (Eq.2)

$\frac{A}{A_{tot}}=1-\left(\frac{A_{1}}{\pi r^{2}}+\frac{A_{2}}{\pi r^{2}}\right)$ (Eq.3)

$A_{1}=R^{2}\cos^{-1}\left(1-\frac{H}{R}\right)-\left(R-H\right)\sqrt{2RH-H^{2}}$(Eq.4)

$A_{2}=r^{2}\cos^{-1}\left(1-\frac{h}{r}\right)-\left(r-h\right)\sqrt{2rh-h^{2}}$ (Eq.5)

Writing $b$ in the small circle ($r$,$h$) and the large circle ($R$,$H$) gives the relation:

$b=\sqrt{2RH-H^{2}}=\sqrt{2rh-h^{2}}$ (Eq.6)

$\frac{A}{A_{tot}}=1-\frac{1}{\pi r^{2}}\left(R^{2}\cos^{-1}\left(1-\frac{H}{R}\right)+r^{2}\cos^{-1}\left(1-\frac{h}{r}\right)-\sqrt{2RH-H^{2}}\left(R-H+r-h\right)\right)$ (Eq.7)

$h$ and $H$ can be written as a function of ($r$,$R$ and $\delta$) using Pythagoras relation in the triangles OMN and CMN:

$r^{2}=(H-\delta)^{2}+\left(\frac{b}{2}\right)^{2}$ (Eq.8)

$R^{2}=(R-H)^{2}+\left(\frac{b}{2}\right)^{2}$ (Eq.9)

Subtracting the two expressions gives:

$H=\frac{r^{2}-\delta^{2}}{2\left(R-\delta\right)}$ (Eq.10)

Moreover, as

$\delta=h+H-r$ (Eq.11)

then

$h=\delta+r-\frac{r^{2}-\delta^{2}}{2\left(R-\delta\right)}$ (Eq.12)

The expression of the ratio $\frac{A}{A_{tot}}$ can be then derived as a function of ($r$,$R$,$\delta$).

A series expansion at the first order of $\frac{A}{A_{tot}}\left(r,R,\delta\right)$ in $\delta$ when $\frac{A}{A_{tot}}\left(r,R,\delta\right)\to\frac{1}{2}$ is done:

$\begin{matrix} \frac{A}{A_{tot}}\left(r,R,\delta\right)=\frac{\pi r^{2}+\sqrt{4r^{2}R^{2}-r^{4}}-2R^{2}\cos^{-1}\left(1-\frac{r^{2}}{2R^{2}}\right)-2r^{2}\cos^{-1}\left(\frac{r}{2R}\right)}{2\pi r^{2}} \\ -\frac{r^{5}-6r^{3}R^{2}+8rR^{4}+2R^{2}\sqrt{4R^{2}-r^{2}}\sqrt{4r^{2}R^{2}-r^{4}}}{\pi r^{2}R\left(4R^{2}-r^{2}\right)^{\frac{3}{2}}}\delta+O\left[\delta\right]^{2} \end{matrix}$ (Eq.13)

and $\delta\left(r,R\right)$ is determined for $\frac{A}{A_{tot}}=\frac{1}{2}$:

$\delta\left(r,R\right)=-\frac{R\left(4R^{2}-r^{2}\right)^{\frac{3}{2}}\left(2R^{2}\cos^{-1}\left(1-\frac{r^{2}}{2R^{2}}\right)+2r^{2}\cos^{-1}\left(\frac{r}{2R}\right)-\pi r^{2}-\sqrt{4r^{2}R^{2}-r^{4}}\right)}{2\left(r^{5}-6r^{3}R^{2}+8rR^{4}+2R^{2}\sqrt{4R^{2}-r^{2}}\sqrt{4r^{2}R^{2}-r^{4}}\right)}$ (Eq.14)

Assuming $R>>r$, a series expansion at the first order of $\delta\left(r,R\right)$ when $R\to\infty$ gives a simple expression:

$\delta_{Comp}=\frac{r^{2}}{6R}$ (Eq.15)

$\delta_{Comp}=\frac{r^{2}}{6}\kappa$ (Eq.16)

# The chord model: a simple geometric construction.

#
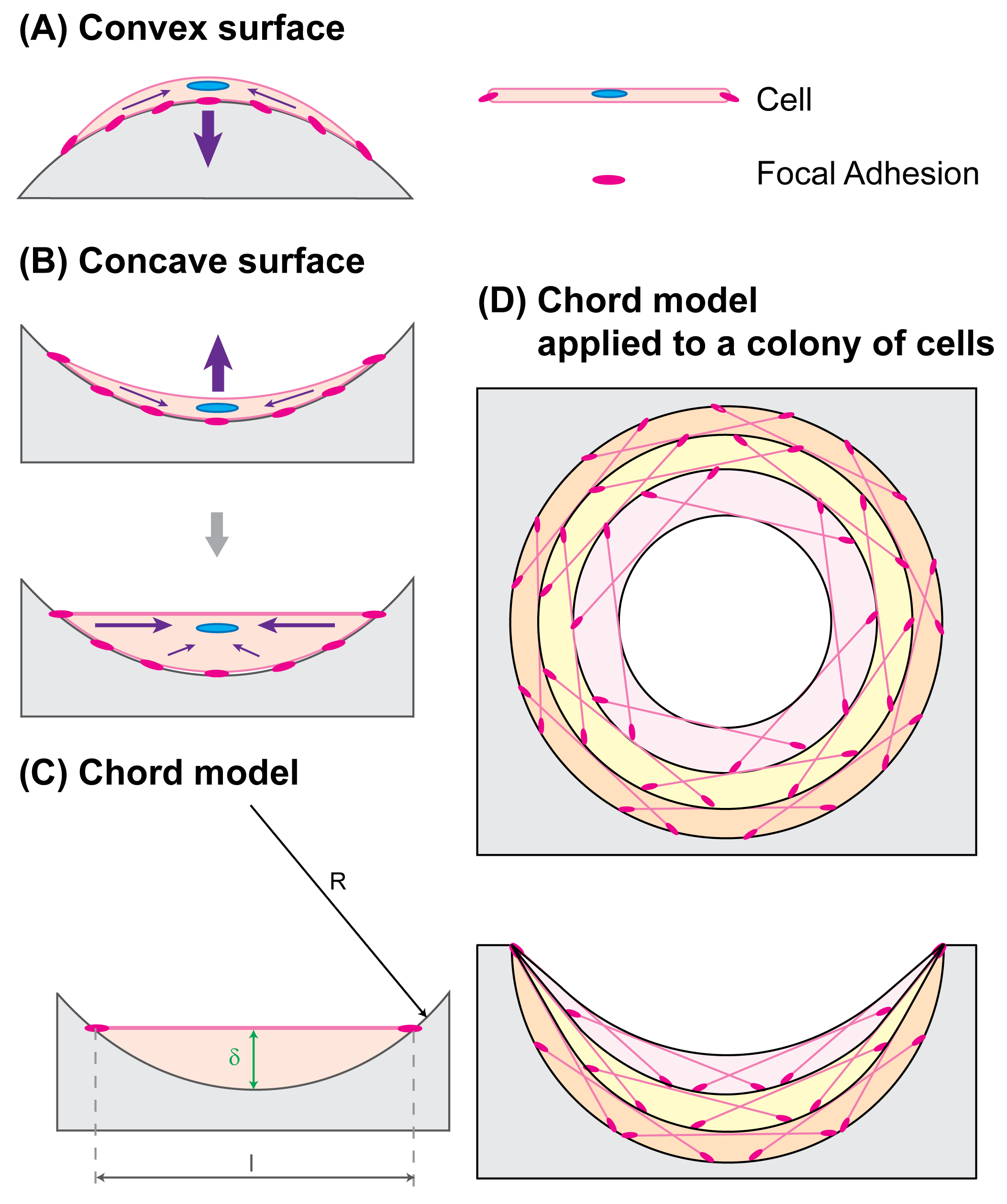


FIGURE S2.

A simple geometrical construction can explain the evolution of the interface observed in in-vitro experiments.

A chord is representing a cell of size $l$ attached to a surface with a local radius of curvature $R$

$R^{2}=\left(\frac{l}{2}\right)^{2}+\left(R-\delta\right)^{2}$ (Eq.17)

$\delta\left(l,R\right)=R-\sqrt{R^{2}-\left(\frac{l^{2}}{4}\right)}$ (Eq.18)

$\delta\left(l,R\right)=R\left(1-\sqrt{1-\left(\frac{l^{2}}{4R^{2}}\right)}\right)$ (Eq.19)

Assuming that $R>>l$, a series expansion at the first order of $\delta\left(l,R\right)$ when $R\to\infty$ gives:

$\delta\left(l,R\right)=R\left(1-1+\frac{1}{2}.\left(\frac{l^{2}}{4R^{2}}\right)\right)$(Eq.20)

$\delta\left(l,R\right)=\frac{l^{2}}{8R}$ (Eq.21)

# $\delta_{Chord}=\frac{l^{2}}{8}\kappa$ (Eq.22)

# The equivalence.

Equations 16 and 22 show that CCTG is a direct consequence of a simple geometric assembly of tensile elements or chords. The quantitative equivalence is highlighted in Fig.S3 by comparing the interfacial motion predicted by the two methods.


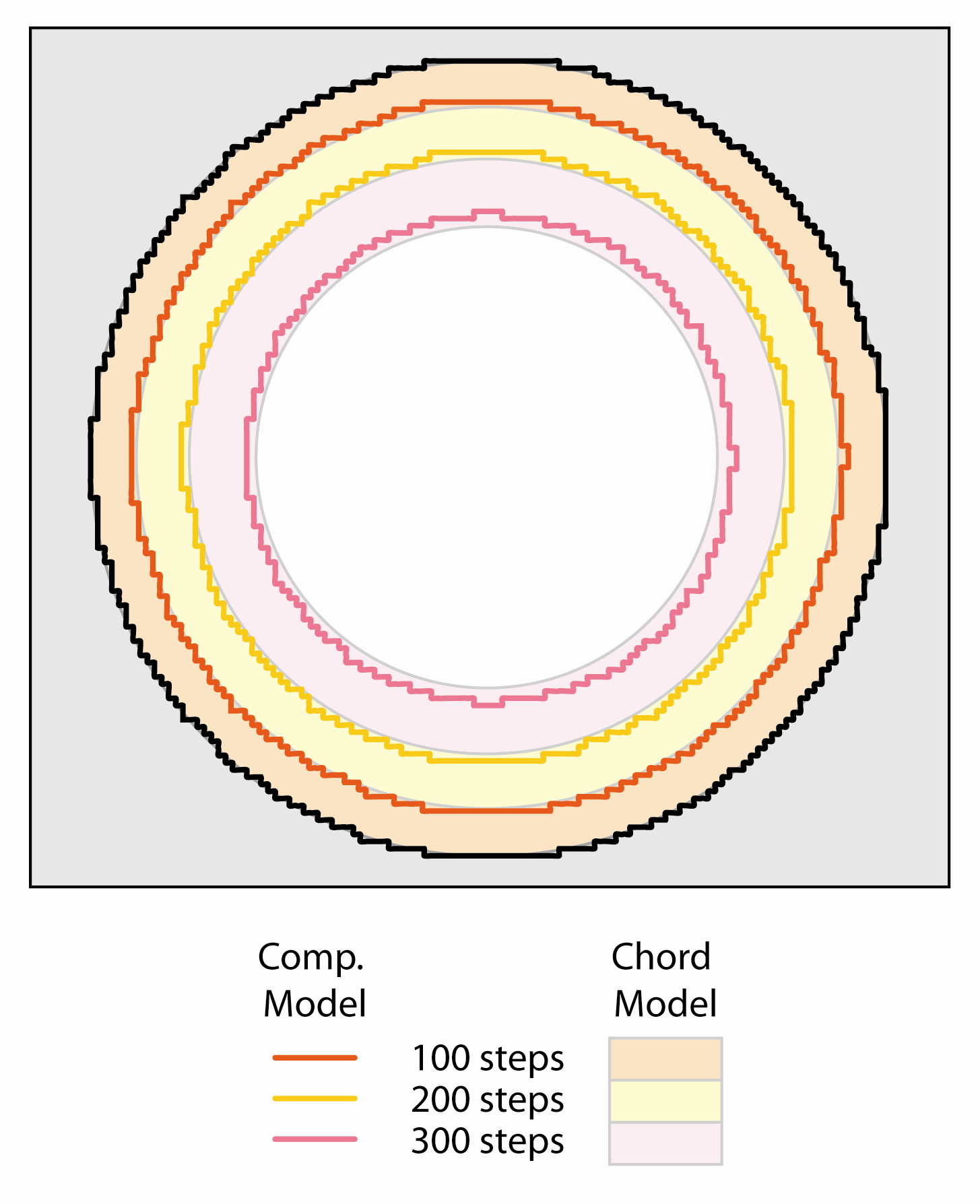


FIGURE S3.

The CCTG description is equivalent to the chord model if the radius of the mask is $r=\frac{\sqrt{3}}{2}l$. The interfaces derived from both models after 100, 200 and 300 iterations are shown as coloured lines for CCTG and grey lines delimiting coloured regions for the chord model (the radius $R(\tau)$being calculated analytically with $r=8.5pxl$, $l=0.047mm$).
